# Inhibition of Hippocampal Matrix Metalloproteinase-3 and -9 Disrupts Spatial Memory

**DOI:** 10.1155/2007/73813

**Published:** 2006-12-14

**Authors:** John W. Wright, Travis E. Brown, Joseph W. Harding

**Affiliations:** ^1^Department of Psychology, Washington State University, Pullman, WA 99164-4820, USA; ^2^Department of Veterinary and Comparative Anatomy, Pharmacology and Physiology, Washington State University, Pullman, WA 99164-6520, USA; ^3^Programs in Neuroscience and Biotechnology, Washington State University, Pullman, WA 99164-6520, USA

## Abstract

Memory consolidation requires synaptic reconfiguration dependent upon extracellular matrix (ECM) molecules interacting with cell adhesion molecules. Matrix metalloproteinase (MMP) activity is responsible for transient alterations in the ECM that may be prerequisite to hippocampal-dependent learning. In support of this hypothesis we have measured increases in MMP-3 and MMP-9 levels within the hippocampus and prefrontal cortex during Morris water maze training. The present investigation extends these findings by determining that infusion of an MMP inhibitor (FN-439) into the dorsal hippocampus disrupted acquisition of this task. In vitro fluorescence enzyme assays to determine the specificity of FN-439 against the catalytic domains of MMP-3 and MMP-9 indicated mean ± SEM IC_50_s of 16.2 ± 7.8 and 210.5 ± 37.8 *μ*M, respectively, while in situ zymography using hippocampal sections treated with FN-439 indicated significant reductions in MMP gelatinase activity. These results suggest that compromising the ability of the dorsal hippocampus to reconfigure ECM molecules by inhibiting MMP activity interferes with appropriate spatial memory acquisition, and support a role for hippocampal MMPs in the phenomena of spatial memory acquisition and storage.

## 1. INTRODUCTION

Extracellular matrix (ECM) molecules mediate changes in the brain's synaptic architecture thought to be critical to the processes of neural plasticity, learning, and memory [1–3]. The ECM is composed of secreted glycoproteins and proteoglycans that form a network to which neurons and glia adhere. The interaction among cells and the ECM is dependent upon several types of cell adhesion molecules (CAMs) including integrins, cadherins, and neural cell adhesion molecules (see [4, 5] for reviews). ECM molecules make possible a wide range of signaling designed to influence cellular proliferation, growth, movement, synaptic stabilization, and apoptosis (see [6, 7] for reviews).

Matrix metalloproteinases (MMPs) are a family of proteinases important to the maintenance and restructuring of the ECM [8–10]. MMPs modulate growth cone extension, neurite development, synaptic transmission and long-term modification, and neuronal degeneration [6, 11–14]. These processes are essential to successful neural plasticity. MMP activity is kept in check by tissue inhibitors of metalloproteinases (TIMPs) that form tight noncovalent complexes with them, thus preventing enzymatic activity [1, 15, 16]. MMP-9 is involved in the remodeling accompanying kainic acid-induced epileptogenesis [17, 18], and deafferentation-induced sprouting in the dentate gyrus [19].

The potential contribution of ECM remodeling to learning and memory has only recently been addressed [5, 6, 20–24]. Our laboratory has focused on hippocampal mediated spatial memory and measured elevations in hippocampal MMP-3 and MMP-9 during acquisition of the Morris water maze task [25, 26]. The MMP inhibitor (MMPi) FN-439 interfered with the late-phase of long-term potentiation (LTP), and when infused intracerebroventricularly (icv) disrupted the acquisition of the Morris water maze task [27].

The present investigation further evaluated the potential role of MMPs in spatial learning. The hippocampus has been shown to mediate the acquisition of spatial memory [28–32], and the dorsal hippocampus is especially important in this regard [33, 34]. Thus, we bilaterally infused FN-439 into the dorsal hippocampus in an attempt to disrupt performance on the Morris water maze task over 8 days of training. Members of an additional group of animals were icv infused with FN-439 for comparison. The following specific questions were addressed. (1) Can bilateral infusions of an MMPi into the dorsal hippocampus block the acquisition of a spatial learning task? (2) Is the magnitude of hippocampal MMPi-induced interference with the acquisition of this task equivalent with that of icv delivered MMPi? (3) How specific is FN-439 against MMP-3 and MMP-9?

## 2. EXPERIMENTAL PROCEDURES

### 2.1. Animals and surgical protocol

The protocols utilized in this investigation minimized pain and discomfort, were approved by the Washington State University Institutional Animal Care and Use Committee, and conformed to the guidelines for the care and use of laboratory animals as required by the National Institutes of Health Guide for the Care and Use of Laboratory Animals (NIH Publication No. 80-23). Male Sprague-Dawley rats (300–350 g, breeding stock derived from Taconic, Germantown, NY) were adapted to a 12-hour light/dark cycle initiated at 0600 hour in the American Association for the Accreditation of Laboratory Animal Care approved vivarium at a temperature of 21 ± 1°C. The animals were housed in pairs and provided with water and food (Harlan Teklad F6 Rodent Diet, Madison, WI) ad libitum except the night prior to surgery when food was withheld. Each animal was prepared with bilateral guide cannulas targeting either the dorsal hippocampi (flat skull coordinates relative to bregma: post: −4.0 mm, lat: ±2.5 mm from midline) or lateral ventricles (post: 1.0 mm, lat: ±1.5 mm from midline) under ketamine-xylazine anesthesia (100 and 2 mg/kg, resp., intramuscularly). The guide was constructed from PE-60 tubing (Clay Adams, Parsippany, NJ) with a heat bulge that rested on top of the cranium thus serving as a stop to further penetration. The total length of the guide was 2.5 cm with a distance from the beveled tip to the heat bulge of 2.0 and 2.5 mm for the dorsal hippocampus and icv, respectively. All infusions were made using a 30-gauge stainless-steel tubing injector with a beveled end that protruded 2.0 mm beyond the tip of the PE guide cannula. The injector was attached to a 10 *μ*L Hamilton syringe by PE-20 tubing. The animals were hand gentled for 5 minutes per day during the 7 days of recovery from surgery. Those animals prepared with dorsal hippocampus guide cannulas were randomly assigned to one of two groups (8 rats each) that received either FN-439 or artificial cerebrospinal fluid vehicle (aCSF; in mM: 124 NaCl, 3 KCl, 1.24 KH_2_PO_2_, 1.3 MgSO_4_, 2.0 CaCl_2_, 26 NaHCO_3_, and 10 D-glucose). Those animals prepared with icv cannulas were similarly assigned.

### 2.2. MMP inhibitor

Reeves et al. [19] determined that a 7.2 mM stock of FN-439 (4-Abz-Gly-Pro-D-Leu-D-Ala-OH, mw = 490.6; MMP inhibitor 1 #444250, Calbiochem, San Diego, Calif) in aCSF infused over 30 minutes to a total volume of 100 *μ*L (350 *μ*g), inhibited MMP-9 activity by 83.6% as compared with aCSF infused control animals. Our laboratory [27] previously used a 7.2 mM stock in aCSF icv infused over 5 minutes to a total volume of 10 *μ*L (35 *μ*g) 10 minutes prior to behavioral testing, and again 3 hours post-testing (cumulative dose = 70 *μ*g). Presently, we utilized a 14.4 mM stock in aCSF infused over 1 minute to a total volume of 5 *μ*L, that is, 2.5 *μ*L for each side (17.5 *μ*g in 2.5 *μ*L aCSF/min) 20 minutes prior to behavioral testing (5 trials), and again 10 minutes after testing (cumulative dose = 70 *μ*g) each day. The protocol used for icv infusion was 35 *μ*g in 5 *μ*L aCSF infused over 1 minute, 20 minutes prior to testing and again 10 minutes following testing (cumulative dose = 70 *μ*g). Behavioral testing in the Morris water maze task was conducted by an experimenter blind to the treatment of each animal.

### 2.3. Morris water maze task

The Morris water maze task [30] was used to test spatial memory acquisition. This protocol has been described in detail [35]. Briefly, each trial entailed placing the animal into the water facing the wall of the pool (1.6 m in diameter painted black, filled to a depth of 30 cm) at one of four locations (North (N), South (S), East (E), and West (W)) and tracking its swimming path and duration (Chromatrac; San Diego Instruments, San Diego, Calif) until the submerged platform was found (12 cm in diameter painted black, 2 cm below the surface). Swim speed was determined from these values. If the animal located the platform within 120 seconds it was permitted 30 seconds on the platform before the next trial commenced. If the animal did not find the platform it was placed on the platform and allowed a 30-second rest period. The animal's entry point was randomized on each trial, and the location of the platform was randomly assigned to one of the four quadrants and remained fixed for each animal throughout training (5 trials/day for 8 days). At the conclusion of the 5 acquisition trials on day 8 each animal was administered one probe trial. During this probe trial (120 seconds) the platform was removed and the time spent within the target quadrant, and the number of crossings into the target quadrant, were recorded.

We also compared the groups concerning positive thigmotaxis on day 8 of training. Since the duration of each of the 5 trials on day 8 depended on how quickly the rat located the platform, the time spent next to, or near (within 20 cm), the wall of the maze was divided by total time of the trial. A ratio was determined for each of the 5 trials and the mean of these five ratios was calculated for each animal and submitted to one-way ANOVA.

### 2.4. Histological examination

Once behavioral testing was completed each animal prepared with hippocampal cannulas was deeply anesthetized using Equithesin (pentobarbital: 100 mg/kg, IP, Jensen-Salsbury Labs, Kansas City, Mont) and transcardally perfused with 0.15 M NaCl followed by 10% formalin. Brains were removed and stored in a 30% sucrose in 10% formalin solution at 4°C for at least 7 days. Each brain was then horizontally sectioned through the hippocampus (40 *μ*m) using a freezing microtome (Spencer Lens, Buffalo, NY). The sections were mounted on gelatin-coated slides and stained with Cresyl violet. The slides were cover-slipped and viewed using an overhead slide projector (Model X-1000, Ken-A-Vision, Raytown, Mont), thus permitting localization of the guide cannula and injector tracts using Paxinos and Watson's atlas [36]. The location of the tip of the injector ranged from −3.6 to −4.5 mm posterior to bregma, lateral 2.2 to 3.0 mm, and ventral to the surface of cortex 2.2–2.8 mm.

Those animals prepared with icv cannulas were anesthetized and injected with 5 *μ*L of fast green dye. The brain was extracted and ventricles were checked for dye. All cannulas were appropriately placed.

### 2.5. MMP enzyme assay

Enzyme assays for MMP activity were conducted in black 96-well plates according to manufacturer instructions (Biomol International LP, Plymouth Meeting, Pa). Catalytic domains for MMP-3 and -9 were used with the fluorogenic peptide substrate: Mca-Pro-Leu-Gly-Leu-Dpa-Ala-Arg-NH_2_. Solutions were preincubated with FN-439 for 30 minutes at 37°C. Subsequently, the fluorogenic peptide substrate was added and fluorescence was measured at 10-minute intervals for one hour using a Perkin-Elmer plate reader. Slopes were derived from the linear portion of the curves and background slopes were subtracted from all samples. The slopes were normalized as percent of enzyme activity compared with activity in the absence of inhibitor. IC_50_ values were extrapolated using quadratic and sigmoidal curve fit programs for each sample and were averaged.

### 2.6. In situ zymography

Naïve rats were euthanized by decapitation, their brains were sectioned (10 *μ*m) using a cryostat and the sections were mounted on slides and stored at −80°C for no longer than a week. These sections were warmed to room temperature for 10 minutes and preincubated for 3 hours in either PBS (pH 7.4 control) or 14.4 mM FN-439 in PBS at 37°C. The control slides were then incubated for an additional hour with DQ-gelatin-FITC (DQ, Molecular Probes, Eugene, Ore) and the treated slides were incubated with DQ containing 14.4 mM FN-439 for 1 additional hour. Following incubation, the slides were washed in PBS, fixed with 4% paraformaldehyde in PBS, and cover slipped using prolong antifade mounting medium containing DAPI (Molecular Probes, Eugene, Ore). The slides were examined using a Zeiss Axioplan 2I microscope using epifluorescent illumination and appropriate filter sets for visualizing DQ (FITC) and DAPI (UV) fluorescence. Images were captured at 100–200X magnification using a Kodak DC290 digital camera and Photoshop software (Adobe Systems Inc, San Jose, Calif).

### 2.7. Statistical analyses

The mean latencies and distances to find the platform and swim speeds during each daily block of five trials in the water maze were analyzed using Groups X Days ANOVAs, with repeated measures on the second factor. One-way ANOVAs were utilized to test for potential differences among the groups on days 1 and 8 of acquisition, to compare levels of thigmotaxis (swimming near the walls of the maze) on day 8 of training, and regarding time spent within, and the number of crossings into, the target quadrant during probe trials. All significant effects from ANOVA analyses were further evaluated using Newman-Keuls post hoc tests with the level of significance set at *P* < .05.

## 3. RESULTS

### 3.1. Morris water maze performance

**Figure 1:**
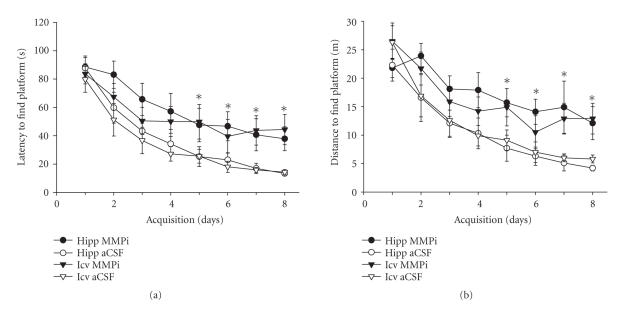
Mean ± SEM group changes in latencies (a) and distances (b) to locate the platform in the Morris water maze during 8 acquisition days. Five trials were administered per day with the location of the platform fixed during training for each animal. Bilateral injections of an MMP inhibitor (MMPi: FN-439) into dorsal hippocampi (Hipp) or intracerebroventricularly (icv) significantly interfered with the acquisition of the task. In contrast, bilateral injections of the vehicle, artificial cerebrospinal fluid (aCSF), into dorsal hippocampi or icv did not interfere with the acquisition of this task of spatial memory. Those groups injected with MMPi revealed significantly longer latencies to find the platform than the aCSF injected groups on days 5–8 of acquisition. *P < .05.

Figure 1 presents the mean ± SEM latencies (panel (a)) and distances swum (panel (b)) to find the submerged platform for each group over the 8 days of acquisition training. These groups were not different regarding latencies to find the platform at the initiation of training on day 1 (F_3,28_ = 0.27, *P* > .10). However, the overall results from the 4 (groups) ×8 (days) ANOVA indicated that both groups treated with MMPi evidenced impaired acquisition as compared with aCSF infused groups (F_3,28_ = 2.97, *P* < .05). There was also an expected days effect (F_7,196_ = 58.41, *P* < .0001); however, the interaction was not significant (F_21,196_ = 1.04, *P* > .10). Post hoc analyses of the days effect indicated that latencies on days 5–8 were less than those on days 1–3, and days 3 and 4 were less than days 1 and 2 of acquisition. There were group differences on day 8 of acquisition (F_3,28_ = 8.95, *P* < .001). Those animals injected with MMPi into the dorsal hippocampus or icv required significantly longer search times than those that received aCSF into the hippocampus or icv (day 8 mean ± SEM latencies: 37.9 ± 8.4, 44.4 ± 10.7, 13.4 ± 1.0, and 14.3 ± 1.9 s, resp.).

One-way ANOVA indicated that the groups were not different on day 1 of acquisition regarding distances swum to find the platform (F_3,28_ = 0.84, *P* > .10; Figure 1(b)). The 4 (groups) ×8 (days) ANOVA revealed a groups effect (F_3,28_ = 3.54, *P* < .05), a significant days effect (F_7,196_ = 9.89, *P* < .0001), but no interaction (F_21,196_ = 1.15, *P* > .10). Post-hoc analyses of the groups effect indicated that both groups given MMPi swam greater distances to find the platform than those injected with aCSF. Post hoc analyses of the days effect suggested that distances on days 4–8 were less than those of days 1 and 2. Finally, there were group differences on day 8 (F_3,28_ = 7.08, *P* < .005). Those rats injected with MMPi into the hippocampus or icv swam significantly longer distances to find the platform than those given aCSF into the hippocampus or icv (12.1 ± 2.9, 12.9 ± 2.7, 4.2 ± 0.5, and 5.8 ± 0.7 m, resp.).

**Figure 2:**
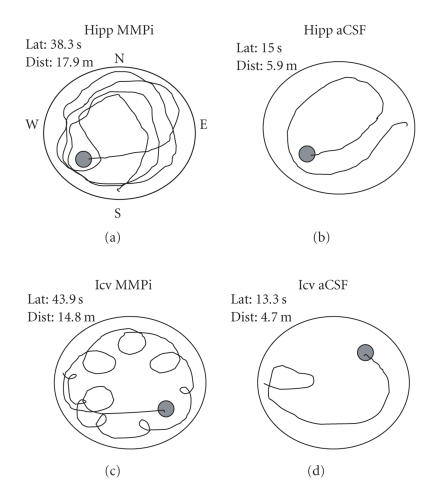
Representative examples of search patterns in the Morris water maze during a 120-second trial on day 8 of acquisition training by one member of each group. Latency (Lat) and distance (Dist) to find the platform are provided for each rat. Bilateral injection of MMPi into dorsal hippocampi (Hipp) or icv resulted in impaired search patterns characterized by frequent swimming near the walls of the maze (positive thigmotaxis). Those animals injected with aCSF into dorsal hippocampi or icv displayed superior search patterns culminating in short latencies and distances to find the platform. Members of these groups did not differ with respect to swim speeds which ranged from 0.30 to 0.32 m/s.

Those rats injected with aCSF displayed superior search strategies as compared with members of both MMPi treated groups (Figure 2). This was illustrated by group differences in positive thigmotaxis, that is, swimming at or near the walls of the maze (F_3,28_ = 9.09, *P* < .001). Post hoc analyses indicated that those animals infused with MMPi into the hippocampus (0.37 ± 0.09) or icv (0.45 ± 0.11) revealed greater thigmotaxic tendencies than those animals infused with aCSF into the hippocampus (0.17 ± 0.06) or icv (0.14 ± 0.05).

**Figure 3:**
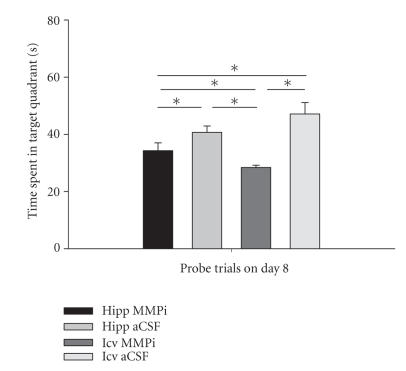
Mean ± SEM time spent by members of each group in the target quadrant during one 120-second probe trial (platform removed) following acquisition training on day 8 in the Morris water maze. Bilateral injection of an MMPi into dorsal hippocampi or icv significantly reduced time spent in the target quadrant as compared with bilateral injection of aCSF. Those animals icv injected with MMPi revealed the least time spent in the target quadrant (28.4 ± 0.8 seconds) followed by those injected with MMPi into the dorsal hippocampi (34.3 ± 2.7 seconds). Those groups injected with the aCSF into the dorsal hippocampi or icv displayed significantly greater spent time (40.7 ± 2.2 and 47.1 ± 4.0 s, resp.). *P < .05.

Results from probe trials conducted at the conclusion of acquisition training on day 8 are presented in Figure 3, and indicated differences among the groups regarding time spent in the target quadrant (F_3,28_ = 9.08, *P* < .001). Post hoc analyses indicated that those animals injected with aCSF into the hippocampus or icv (40.7 ± 2.2 and 47.1 ± 4.0 s, resp.) revealed significantly greater time spent in the target quadrant than those rats treated with MMPi into the hippocampus or icv (34.3 ± 2.7 and 28.4 ± 0.8 s, resp.). The group that received icv MMPi indicated significantly less time in the target quadrant than the other groups. There were no differences considering the number of entries into the target quadrant (F_3,28_ = 0.98, *P* > .10). Nor were there differences in overall swim speeds during the 8 days of training for those groups treated with MMPi into the hippocampus or icv, and those injected with aCSF into the hippocampus or icv (0.32 ± 0.02, 0.31 ± 0.02, 0.30 ± 0.02, and 0.31 ± 0.02 m/s, resp.).

### 3.2. MMP enzyme assay

**Figure 4:**
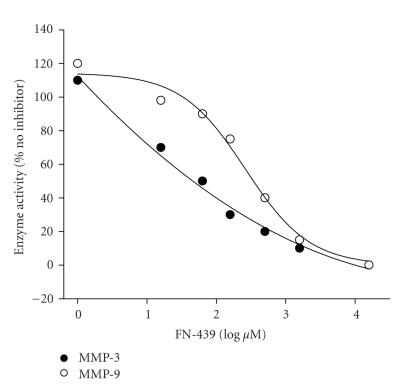
Results from fluorescence enzyme assays to establish the specificity of FN-439 for the catalytic domains of MMP-3 and MMP-9. The slopes were derived from the linear portion of the curves and background slopes were subtracted from all samples. The slopes were normalized as percent of enzyme activity compared with activity in the absence of inhibitor. IC_50_ values were extrapolated using quadratic or sigmoidal curve fit programs for each sample and averaged. The determined mean ± SEM IC_50s_ for MMP-3 and MMP-9 were 16.2 ± 7.8 and 210.5 ± 37.8 *μ*M.

The mean ± SEM IC_50_ values for MMP-3 and -9 were determined to be 16.2 ± 7.8 and 210.5 ± 37.8 *μ*M, respectively, indicating high specificity against MMP-3 (stromelysin-1) and reasonably good specificity for MMP-9 (gelatinase B) (Figure 4). These IC_50_ values were the reverse of those reported by Odake et al. [37], that is, 30 *μ*M against gelatinase and 150 *μ*M against stromelysin. There are at least two possible explanations for these differences. First, Odake and colleagues used HSF stromelysin and HG gelatinase as substrates, while we used MMP-3 (stromelyisin-1) and MMP-9 (gelatinase B). Second, we used only the catalytic domains of MMP-3 and MMP-9.

### 3.3. In situ zymography

**Figure 5:**
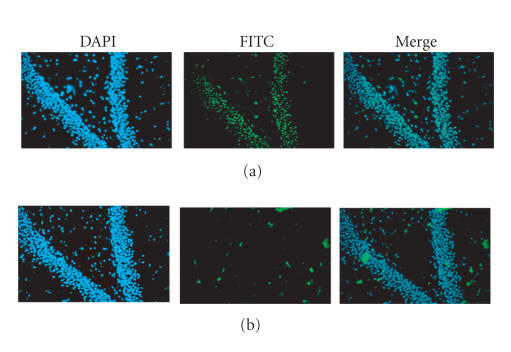
Representative in situ zymography results demonstrating gelatinase activity by MMPs in the dentate gyrus in the presence of (a) PBS (control condition) upper panels or (b) FN-439 (14.4 mM) lower panels. The equivalent blue fluorescence of cell nuclei suggests no cellular toxicity. MMP-2 and MMP-9 activity, visualized as green fluorescence, was significantly inhibited by FN-439 as evidenced by reductions in green fluorescence.

Figure 5 presents the results of incubating hippocampal sections with PBS or 14.4 mM FN-439 on MMPs (green fluorescence) in situ. A similar appearance of cell nuclei (blue fluorescence) was noted comparing control (PBS) and FN-439 treated sections, suggesting no cellular toxicity. The basal activity of MMP-9 was significantly reduced following FN-439 treatment as indicated by a lack of green fluorescence. These in situ results suggest that FN-439 significantly inhibited MMP-2 and MMP-9 in the hippocampus.

## 4. DISCUSSION

The present investigation was designed to further evaluate the ability of an MMP inhibitor to influence acquisition of a spatial memory task. We were particularly interested in determining the specificity of FN-439 and whether infusion of this inhibitor into the dorsal hippocampus disrupted acquisition of a spatial memory task. The in vitro fluorescence assay results demonstrated that FN-439 had a maximal effect upon the MMP-3 catalytic domain and good specificity for the MMP-9 catalytic domain. In situ zymography further determined that FN-439 significantly reduced MMP gelatinase activity in hippocampal sections as compared with PBS controls. In a previous investigation Reeves et al. [19] used a significantly larger icv dose of FN-439 (350 *μ*g) than in the present study (70 *μ*g), following unilateral lesions of the entorhinal cortex resulting in “collateral sprouting of the crossed temporodentate fiber pathway.” These rats were shown to lack the ability to demonstrate LTP in the sprouting pathway. This points to an important role for MMPs in the process of synaptogenesis and the capacity to form LTP in the deafferentation/sprouting model.

Presently, FN-439 significantly interfered with the acquisition of the Morris water maze task of spatial learning and was equivalently effective whether infused into the dorsal hippocampus or icv delivered. Group differences in rate of acquisition could not be attributed to differences in swim speed. Although we have not tested FN-439 for hippocampal penetrability, we have previously shown that icv delivered radio labeled small peptides influence c-fos expression in the hippocampus [38]. It is likely that this low molecular weight inhibitor is also capable of penetrating the hippocampus and influencing signaling when icv injected. There were group differences comparing probe trial results with the aCSF infused groups revealing greater time spent in the target quadrant than the MMPi treated groups. The degree of persistence to enter and remain in the target quadrant has been utilized as a measure of successful acquisition. Although we presently noted MMPi-induced interference with appropriate acquisition of this task a larger dose of this inhibitor would be expected to have an even greater negative impact on performance. Related to this, the daily infusion volume of 2.5 *μ*L over one minute into the dorsal hippocampus may have resulted in tissue damage that could explain the decreased persistence of the group given aCSF to stay in the target quadrant during probe trials as compared with the group given icv aCSF.

Nagy et al. [3], Meighan et al. [27], and the present investigation move this research area towards the establishment of a causal relationship between changes in brain MMP expression and learning. Nagy and colleagues reported that the treatment of hippocampal slices with an MMP-2/9 inhibitor disrupted late-phase LTP but did not affect early-phase LTP. Similar results were obtained using MMP-9 null mutant mice. In agreement with a previous paper from our laboratory [27] the present findings suggest a link between inhibition of hippocampal MMP-3 and -9 and significantly reduced ability to acquire a spatial memory task, and establish an important role for changes in MMP-3 and -9 expression within the dorsal hippocampus in the mediation of spatial memory formation. Thus, these results add further support to earlier work indicating that ECM molecules are intimately involved in the modulation of synaptic remodeling and connectivity [10, 21, 24, 39–42].

In summary, hippocampal MMP-3 and MMP-9 appear to be instrumental in the activation and maintenance of the neural plasticity presumed to underlie spatial learning and memory. In the context of current models of memory encoding and consolidation [7], compromising the ability of the dorsal hippocampus to reconfigure ECM molecules by interfering with MMP activity appears to prevent appropriate memory acquisition, and in turn its ability to pass the stored memory onto other locations (e.g., prefrontal cortex) for long-term storage.
